# Oxidative balance score and all-cause mortality among hypertensive individuals

**DOI:** 10.7189/jogh.15.04285

**Published:** 2025-11-14

**Authors:** Lian-Zhen Huang, Hong-Bin Zhang, Mei Qin, Ze-Bin Ni, Wei-Feng Huang, Ji Li, Li-Ping Sheng, Li-Yun Guo, Jin-Yan Zhang

**Affiliations:** 1 Department of Gastroenterology and Hepatology, The First Affiliated Hospital of Xiamen University, School of Medicine, Xiamen University, Xiamen, China; 2 Department of Physical Examination, The First Affiliated Hospital of Xiamen University, School of Medicine, Xiamen University, Xiamen, China; 3 The School of Clinical Medicine, Fujian Medical University, Fuzhou, China; 4 Xiamen Daytime Medical Management Quality Control Center, Xiamen, China

## Abstract

**Background:**

Oxidative stress contributes to hypertension and its complications. The oxidative balance score (OBS) could therefore provide insight into the relationship with mortality in hypertensive individuals. We aimed to investigate the association between OBS and all-cause mortality using data from National Health and Nutrition Examination Survey 2007–18.

**Methods:**

Our sample comprised 11 196 hypertensive participants. We calculated OBS based on dietary and lifestyle factors and categorised participants accordingly into quartiles. We used Cox proportional hazards model to assess associations between OBS and mortality, and restricted cubic spline (RCS) analyses to determine dose-response relationships. Lastly, we conducted Kaplan-Meier survival curves and stratified/sensitivity analyses.

**Results:**

1764 deaths occurred during a median follow-up time of 73.4 months. Higher OBS was significantly associated with lower mortality risk, whereby participants in the highest OBS quartile had a 34% reduced mortality risk compared to those in the lowest quartile (hazard ratio (HR) = 0.66; 95% confidence interval (CI) = 0.51–0.84, *P* = 0.001). Each unit increase in OBS reduced mortality risk by 2% (HR = 0.98; 95% CI = 0.96–0.99, *P* < 0.001). We observed similar results for dietary and lifestyle OBS. RCS analyses indicated a nearly linear dose-response relationship between OBS and the risk of all-cause mortality (*P*-value for nonlinearity >0.05). Kaplan-Meier curves confirmed better survival in those with higher OBS (log-rank *P*-value <0.001). Stratified analyses showed stronger protective effects in individuals with middle incomes and those without a history of cancer (*P*-value for interaction <0.05). Sensitivity analyses confirmed the robustness of these findings.

**Conclusions:**

Higher OBS levels, along with its dietary and lifestyle subscale scores, are significantly associated with a reduced risk of all-cause mortality among hypertensive individuals. These findings highlight the importance of oxidative balance and the potential benefits of antioxidant-rich diets and healthy lifestyles in reducing mortality risk for this population.

Hypertension affects over one billion adults worldwide, including approximately 116 million in the USA, and remains a leading contributor to global morbidity and mortality [1]. As a major risk factor for cardiovascular disease (CVD) events such as coronary heart disease, heart failure, and stroke, it is responsible for nearly 10 million deaths annually and accounts for about 10% of global healthcare expenditures [1–3]. The primary causes of death among hypertensive individuals include heart attacks, heart failure, stroke, kidney disease, vascular diseases, metabolic syndrome, retinopathy, and aortic dissection [4,5]. Importantly, hypertension-related mortality is strongly influenced by modifiable lifestyle factors, including unhealthy diets, obesity, smoking, excessive alcohol consumption, and physical inactivity [6,7].

The pathogenesis of hypertension is multifactorial, with a growing body of evidence highlighting the critical role of oxidative stress [8–10]. Oxidative stress results from an imbalance between pro-oxidant and antioxidant forces, leading to elevated levels of reactive oxygen species (ROS) which, in hypertension, can cause endothelial dysfunction through lipid peroxidation and inflammation, promote vascular constriction and remodelling, and disrupt the regulation of vasoactive substances such as angiotensin II and endothelin-1 [11–14]. These mechanisms contribute to increased blood pressure and end-organ damage.

Given the importance of oxidative stress, the oxidative balance score (OBS) has been developed to effectively assess the overall balance between antioxidant and pro-oxidant exposures from both diet and lifestyle. It incorporates various factors such as dietary intake of antioxidants (*e.g.* vitamins C and E, carotenoids), pro-oxidants (*e.g.* saturated fat, iron), and lifestyle behaviours associated with hypertension risk, such as smoking, alcohol consumption, physical activity, and body mass index (BMI) [15]. A higher OBS indicates a more favourable antioxidant status, which in turn reflects a lower risk of mortality through mechanisms such as improved vascular function and reduced CVD risk.

Previous research has shown that a higher OBS is associated with a reduced risk of various chronic diseases, including cancer [16], CVD [17], and non-alcoholic fatty liver disease [18]. While some studies have suggested a link between OBS and a lower risk of developing hypertension, they were often methodologically limited, as they either adopted cross-sectional designs or have not specifically investigated the relationship between OBS and long-term mortality in individuals with hypertension [19]. A deeper investigation of this association could therefore help us understand the importance of lifestyle modifications in this high-risk population. With this in mind, we utilised the National Health and Nutrition Examination Survey (NHANES) database to investigate the association between OBS and all-cause mortality in US adults with hypertension. We hypothesise that a higher OBS is associated with lower all-cause mortality in adults with hypertension.

## METHODS

### Study design and population

The NHANES, conducted by the National Center for Health Statistics (NCHS), assesses the health and nutritional status of US adults and children in two-year cycles through robust data collection that extends beyond questionnaire-based interviews to include physical examinations and laboratory analysis of biospecimens [20]. The NHANES protocol received ethical approval from the NCHS Ethics Review Board, and written informed consent was obtained from all participants. Given that we utilised deidentified, publicly available data from the NHANES database, it was deemed exempt from institutional review board oversight. We reported our findings per the STROBE guidelines [21], while our analysis of a subset of the NHANES dataset aligns with the Journal of Global Health’s Guidelines for Reporting Analyses of Big Data Repositories Open to the Public (Table S4 in the **Online Supplementary Document**).

We identified 34 770 individuals aged 20 years and older from the initial 59 842 participants in NHANES 2007–18. We excluded those without hypertension (n = 19 914), those with missing survival status (n = 32), those with incomplete OBS data (n = 2444), and those with missing covariate data (n = 1184). We found no overlap between missing OBS or covariate data and survival data. Ultimately, we retained 11 196 participants in the final analysis (Figure S1 in the **Online Supplementary Document**).

### Definition of hypertension

We defined hypertension according to the 2020 global hypertension practice guidelines from the International Society of Hypertension, ensuring consistency with current clinical standards and facilitating comparability with recent studies. Specifically, participants were considered hypertensive if they met at least one of the following criteria: an average systolic blood pressure (SBP)≥140 mm Hg; an average diastolic blood pressure (DBP)≥90 mm Hg, both based on at least three measurements; self-reported physician diagnosis of hypertension; or current use of antihypertensive medication [22,23].

### Calculation of OBS

The OBS integrated scores from 16 nutrients and 4 lifestyle factors, including 5 pro-oxidants and 15 antioxidants [24]. The 16 nutrients were dietary fibre, carotenoids, riboflavin, niacin, vitamin B6, total folate, vitamin B12, vitamin C, vitamin E, calcium, magnesium, zinc, copper, selenium, total fat, and iron. We derived these data from two 24-hour dietary recall interviews and categorised all elements into three distinct groups based on their weighted distribution. The four lifestyle factors included physical activity, BMI, alcohol consumption, and smoking (determined by cotinine levels in the blood). Among these, total fat, iron, BMI, alcohol consumption, and smoking were considered pro-oxidants, while the remaining factors were classified as antioxidants. We scored antioxidant factors from 0 to 2, and pro-oxidant factors from 2 to 0. Using alcohol intake data, also collected from two 24-hour dietary recall interviews, we categorised individuals into three groups: heavy drinkers (≥15 g/d for women and ≥30 g/d for men), non-heavy drinkers (0–15 g/d for women and 0–30 g/d for men), and non-drinkers, which were assigned scores of 0, 1, and 2, respectively. We calculated BMI by dividing body weight by height squared and classified individuals into three categories: normal weight (BMI<25 kg/m^2^), overweight (25≤BMI<30 kg/m^2^), and obese (BMI≥30 kg/m^2^), with scores assigned as 2, 1, and 0, respectively [25]. NHANES employed the Physical Activity Questionnaire to assess participants' weekly involvement in vigorous and moderate-intensity physical activities, as well as their exercise and leisure activities, capturing both the frequency and duration of these engagements. We aggregated the total activity levels from these three domains and translated them into metabolic equivalent of tasks (METs) levels, based on which we categorized participants into low (<500 MET-minutes/week), moderate (500–1000 MET-minutes/week), and high (>1000 MET-minutes/week) MET groups [26], with corresponding scores of 0, 1, and 2, respectively. We calculated an individual’s OBS by summing all component scores, with higher scores indicating greater antioxidant exposure. OBS components were scored using established criteria (Table S1 in the **Online Supplementary Document**).

### Ascertainment of mortality

We downloaded data from a dataset managed by the NCHS of the US Centers for Disease Control and Prevention [27], which links mortality information to participants from the NHANES and the National Health Interview Survey. The follow-up period began on the date participants underwent the baseline survey and ended either on the date of death or the most recent update of the linked National Death Index database (31 December 2019).

### Covariates

We included covariates that could confound the relationship between oxidative balance and mortality based on previous research and biological plausibility. Specifically, we adjusted for sociodemographic variables (age, sex, race/ethnicity, education level, marital status, and poverty income ratio (PIR)), lifestyle factors (*e.g.* smoking), and comorbid conditions (*e.g.* history of CVD, history of cancer, hyperlipidaemia, diabetes mellitus) to minimise potential residual confounding. We also included total energy intake to account for overall diet quantity. We also formed the following categories:

– Race/ethnicity: Mexican American, other Hispanic, non-Hispanic White, non-Hispanic Black, or other races.

– Education levels: high school or below, some college or associate degree, and college graduate or above.

– Marital status: married/living with partner, widowed/divorced/separated, and never married.

– Smoking status: never smokers (less than 100 cigarettes in their lifetime), former smokers (more than 100 cigarettes in their lifetime, but currently abstaining), and current smokers (more than 100 cigarettes in their lifetime and currently smoking).

Diabetes mellitus was diagnosed when at least one of the following criteria was met: a previous diagnosis of diabetes by a physician, fasting glucose ≥126 mg/dL, glycated haemoglobin (HbA1c)>6.5%, and use of diabetes medication or insulin. Hyperlipidaemia was defined as having at least one of the following: total cholesterol ≥200 mg/dL, triglycerides ≥150 mg/dL, low-density lipoprotein cholesterol ≥130 mg/dL, or high-density lipoprotein cholesterol <40 mg/dL. History of CVD included conditions such as heart attack, angina, coronary heart disease, or stroke.

### Statistical analysis

All analyses accounted for the complex survey design through the incorporation of sample weights, stratification, and clustering. We presented continuous variables as weighted means with standard errors (SEs), and categorical variables as weighted percentages with corresponding 95% confidence intervals (CIs).

Following NHANES guidelines [28], we combined survey cycle sample weights by dividing the two-year weights by six. We assessed OBS levels as both a continuous variable (incremental per unit) and a categorical variable (divided into quartiles). We compared the participants’ baseline characteristics using weighted one-way analysis of variance (ANOVA) for continuous variables and weighted Rao-Scott χ^2^ test for categorical variables. The relationship between OBS and mortality risk among hypertensive patients was examined using weighted Cox regression models, with results expressed as hazard ratios (HRs) with a 95% CIs. We used three models: model 1 was unadjusted for any covariates; model 2 was adjusted for sex, age, and race/ethnicity; and model 3 was adjusted for all covariates, including sex, age, race/ethnicity, education level, marital status, PIR, smoking status, history of CVD, history of cancer, hyperlipidaemia, diabetes mellitus, and total energy intake. We further differentiated OBS into dietary and lifestyle components to explore their respective associations with mortality risk.

We performed a restricted cubic spline (RCS) analysis with four knots positioned at the 5th, 35th, 65th, and 95th percentiles to investigate the potential nonlinear relationship between OBS and mortality risk in hypertensive patients. Additionally, we conducted stratified analyses based on sex, age groups (20–39 years, 40–59 years, and 60 years or older), race/ethnicity, education level, marital status, PIR categories (<1.30, 1.30–3.50, and ≥3.50), smoking status, history of CVD, history of cancer, diabetes, and hyperlipidaemia. We also performed survival analyses using weighted Kaplan-Meier curves.

We conducted two sensitivity analyses to verify the robustness of our results. Initially, we re-analysed the data after excluding participants who died within the first two years of follow-up (n = 351). We then excluded 634 individuals with implausible energy intake levels (males: <800 kcal/d or >4200 kcal/d; females: <500 kcal/d or >3500 kcal/d).

We used *R*, version 4.3.2 (R Foundation for Statistical Computing, Vienna, Austria) and Free Statistics software, version 1.9.2 (Beijing FreeClinical Medical Technology Co., Ltd, Beijing, China), for all statistical analyses. We used the ‘survey’ package in R for weighted data analysis, employing the ‘svydesign’ function for constructing complex sampling designs, ‘svymean’ for calculating weighted means and percentages, and ‘svycoxph’ for fitting Cox regression models. We employed the Bonferroni correction to account for multiple testing using a two-sided *P*-value of 0.05, which resulted in an adjusted significance level of *P* = 0.017 (0.05/3). A two-tailed *P* < 0.05 indicated statistical significance.

## RESULTS

### Baseline characteristics

Our analytical sample included 11 196 participants (50.15% women (95% CI = 49.11–51.19); weighted mean age = 57.22 (SE = 0.22); 46.87% aged ≥60 years (95% CI = 45.38–48.37)). By race/ethnicity, 5.74% (95% CI = 4.61–7.13) identified as Mexican American, 70.79% (95% CI = 67.78–73.63) as non-Hispanic White, and 12.69% (95% CI = 10.93–14.68) as non-Hispanic Black. We divided the cohort into OBS quartiles (2783 participants in Q1 (≤14), 2445 in Q2 (14–19), 2991 in Q3 (19–25) and 2977 in Q4 (>25)) and compared them by baseline characteristics (**Table 1**). Compared to those in the lowest quartile, participants in higher OBS quartiles were more likely to be male and non-Hispanic White, and also tended to have higher education levels, greater family income, and increased total energy intake. In terms of health-related characteristics, individuals in Q4 had a lower prevalence of CVD history, diabetes mellitus, and all-cause mortality compared to those in Q1, while dietary and lifestyle OBS scores progressively increased across quartiles. There were 525 deaths in OBS Q1, 424 in Q2, 453 in Q3, and 362 in Q4.

**Table 1:** Baseline characteristics of study participants in NHANES 2007–18*

	Total (n = 11 196)	Q1 (≤14, n = 2783)	Q2 (14 to ≤19, n = 2445)	Q3 (19 to ≤25, n = 2991)	Q4 (>25, n = 2977)	*P-*value	*P*-value for trend
**Sex**						0.018	
Male	49.85 (48.81–50.89)	46.62 (44.01–49.25)	50.62 (47.82–53.42)	49.07 (46.82–51.33)	52.22 (49.99–54.45)		0.020
Female	50.15 (49.11–51.19)	53.38 (50.75–55.99)	49.38 (46.58–52.18)	50.93 (48.67–53.18)	47.78 (45.55–50.01)		0.020
**Age in years, x̄ (SE)**	57.22 (0.22)	57.26 (0.38)	57.94 (0.42)	57.33 (0.33)	56.60 (0.32)	0.051	0.042
**Age strata**						<0.001	
20–39	14.31 (13.34–15.33)	15.53 (13.84–17.40)	14.25 (12.56–16.12)	13.47 (11.90–15.22)	14.26 (12.61–16.09)		0.280
40–59	38.82 (37.52–40.14)	35.51 (32.97–38.12)	35.36 (32.50–38.32)	39.33 (36.77–41.95)	42.99 (40.47–45.55)		<0.001
≥60	46.87 (45.38–48.37)	48.96 (46.31–51.61)	50.39 (47.15–53.63)	47.20 (44.52–49.89)	42.75 (40.37–45.16)		<0.001
**Race and ethnicity**						<0.001	
Mexican American	5.74 (4.61–7.13)	5.49 (4.19–7.16)	5.41 (4.10–7.10)	5.86 (4.54–7.52)	6.04 (4.77–7.61)		0.326
Other Hispanic	4.32 (3.54–5.25)	4.49 (3.42–5.87)	4.26 (3.35–5.39)	4.06 (3.16–5.21)	4.47 (3.59–5.55)		0.953
Non-Hispanic White	70.79 (67.78–73.63)	63.61 (59.40–67.62)	71.32 (67.37–74.97)	72.02 (68.77–75.06)	74.22 (71.12–77.10)		<0.001
Non-Hispanic Black	12.69 (10.93–14.68)	20.14 (16.97–23.73)	13.40 (11.24–15.89)	11.01 (9.43–12.81)	8.64 (7.38–10.11)		<0.001
Others	6.46 (5.66–7.36)	6.27 (4.96–7.91)	5.61 (4.54–6.92)	7.05 (5.81–8.54)	6.62 (5.42–8.08)		0.432
**Education level**						<0.001	
High school or less	42.59 (40.81–44.39)	54.44 (51.82–57.03)	45.04 (41.88–48.24)	41.65 (39.12–44.23)	33.68 (30.98–36.50)		<0.001
Some college or associates degree	32.62 (31.24–34.03)	31.21 (28.94–33.57)	32.69 (29.99–35.51)	34.66 (32.31–37.08)	31.69 (29.36–34.11)		<0.001
College graduate or above	24.79 (22.90–26.78)	14.35 (12.40–16.56)	22.27 (19.40–25.43)	23.69 (21.24–26.33)	34.63 (31.71–37.67)		<0.001
**Marital status**						<0.001	
Married/living with partner	64.13 (62.52–65.71)	57.37 (55.04–59.66)	64.77 (62.16–67.30)	64.77 (62.45–67.02)	67.73 (64.71–70.60)		<0.001
Widowed/divorced/separated	25.98 (24.67–27.33)	30.76 (28.73–32.86)	25.97 (23.72–28.35)	27.05 (24.98–29.21)	21.75 (19.72–23.93)		<0.001
Never married	9.89 (9.05–10.81)	11.87 (10.46–13.44)	9.26 (7.88–10.85)	8.19 (6.91–9.67)	10.52 (8.96–12.33)		0.237
**Poverty income ratio, x̄ (SE)**	2.98 (0.04)	2.46 (0.04)	2.89 (0.05)	3.06 (0.05)	3.31 (0.05)	<0.001	<0.001
**Poverty income ratio categories**						<0.001	
<1.3	21.61 (20.14–23.15)	32.35 (30.14–34.65)	22.54 (20.58–24.62)	19.12 (17.13–21.28)	15.89 (14.18–17.76)		<0.001
1.3–3.5	37.07 (35.57–38.59)	39.37 (36.75–42.05)	39.24 (36.73–41.81)	37.57 (35.03–40.18)	33.57 (30.90–36.34)		<0.001
≥3.5	41.32 (39.31–43.37)	28.28 (25.74–30.96)	38.22 (35.34–41.20)	43.31 (40.45–46.23)	50.54 (47.25–53.83)		<0.001
**Smoking**						<0.001	
Never	17.77 (16.72–18.86)	27.24 (25.24–29.33)	17.92 (15.74–20.32)	16.33 (14.46–18.38)	12.50 (11.18–13.95)		<0.001
Former	32.20 (30.91–33.51)	29.30 (27.14–31.56)	32.10 (29.54–34.78)	33.47 (30.89–36.15)	33.08 (31.06–35.17)		0.012
Current	50.04 (48.57–51.51)	43.46 (40.69–46.27)	49.98 (47.10–52.86)	50.20 (47.64–52.77)	54.42 (52.33–56.50)		<0.001
**History of CVD**						<0.001	
No	84.51 (83.58–85.41)	78.71 (76.36–80.89)	84.51 (82.61–86.24)	85.80 (84.08–87.36)	87.31 (85.77–88.70)		<0.001
Yes	15.49 (14.59–16.42)	21.29 (19.11–23.64)	15.49 (13.76–17.39)	14.20 (12.64–15.92)	12.69 (11.30–14.23)		<0.001
**History of cancer**						0.336	
No	84.35 (83.46–85.21)	84.59 (82.67–86.33)	83.53 (81.25–85.58)	85.55 (83.86–87.09)	83.66 (81.94–85.25)		0.798
Yes	15.65 (14.79–16.54)	15.41 (13.67–17.33)	16.47 (14.42–18.75)	14.45 (12.91–16.14)	16.34 (14.75–18.06)		0.798
**Hyperlipidaemia**						0.480	
No	38.75 (37.17–40.34)	37.29 (34.72–39.94)	38.67 (35.91–41.50)	38.43 (35.69–41.24)	40.08 (37.44–42.78)		0.167
Yes	61.25 (59.66–62.83)	62.71 (60.06–65.28)	61.33 (58.50–64.09)	61.57 (58.76–64.31)	59.92 (57.22–62.56)		0.167
**Diabetes mellitus**						<0.001	
No	75.10 (74.04–76.13)	70.52 (68.02–72.91)	72.62 (70.36–74.77)	76.02 (74.16–77.78)	79.08 (77.27–80.79)		<0.001
Yes	24.90 (23.87–25.96)	29.48 (27.09–31.98)	27.38 (25.23–29.64)	23.98 (22.22–25.84)	20.92 (19.21–22.73)		<0.001
**All-cause mortality**						<0.001	
No	87.77 (86.77–88.70)	83.70 (82.00–85.27)	86.35 (84.67–87.87)	88.34 (86.78–89.75)	90.99 (89.55–92.25)		<0.001
Yes	12.23 (11.30–13.23)	16.30 (14.73–18.00)	13.65 (12.13–15.33)	11.66 (10.25–13.22)	9.01 (7.75–10.45)		<0.001
**Total energy intake in kcal/d, x̄ (SE)**	2100.12 (11.15)	1351.28 (16.13)	1774.64 (19.36)	2163.77 (19.36)	2776.44 (27.22)	<0.001	<0.001
**OBS, x̄ (SE)**	20.17 (8.05)	10.40 (0.06)	16.05 (0.04)	21.52 (0.04)	28.42 (0.07)	<0.001	<0.001
**Dietary OBS, x̄ (SE)**	16.02 (0.11)	6.83 (0.06)	12.00 (0.06)	17.43 (0.05)	23.78 (0.07)	<0.001	<0.001
**Lifestyle OBS, x̄ (SE)**	4.14 (0.02)	3.58 (0.04)	4.05 (0.05)	4.09 (0.04)	4.64 (0.03)	<0.001	<0.001

CVD – cardiovascular disease, NHANES – National Health and Nutrition Examination Survey, OBS – oxidation balance score, Q – quartile, SE – standard error, x̄ – mean

*Values are presented as weighted % (95% confidence interval) unless specified otherwise.

### Weighted cox regression analyses

We observed a consistently negative association between OBS, its subscale scores, and the risk of all-cause mortality (**Table 2****)**. In the fully adjusted model 3, individuals in Q4 exhibited a 34% lower mortality risk compared to those in Q1 (HR = 0.66; 95% CI = 0.51–0.84, *P* = 0.001). Each unit increase in OBS was associated with a 2% reduction in mortality risk (HR = 0.98; 95% CI = 0.96–0.99, *P* < 0.001). For dietary OBS, participants in Q4 had a 29% lower mortality risk than those in Q1 (HR = 0.71; 95% CI = 0.57–0.90, *P* = 0.005), with each unit increase being associated with an reduction in the risk of all-cause mortality by 2% (HR = 0.98; 95% CI = 0.97–1.00, *P* = 0.008). For lifestyle OBS, Q4 participants had a 29% lower mortality risk compared to Q1 (HR = 0.71; 95% CI = 0.59–0.84, *P* < 0.001), with each unit increase being associated with an 8% risk reduction (HR = 0.92; 95% CI = 0.88–0.96, *P* < 0.001).

**Table 2:** Associations of OBS and its subscale scores with all-cause mortality in hypertensive individuals*

	Model 1		Model 2		Model 3	
	**HR (95% CI)**	***P*-value**	**HR (95% CI)**	***P*-value**	**HR (95% CI)**	***P*-value**
**OBS**						
OBS	0.97 (0.96–0.98)	<0.001	0.97 (0.96–0.98)	<0.001	0.98 (0.96–0.99)	<0.001
OBS quartile						
*Q1*	ref		ref		ref	
*Q2*	0.82 (0.70–0.95)	0.011	0.77 (0.66–0.90)	<0.001	0.84 (0.71–1.00)	0.057
*Q3*	0.69 (0.59–0.80)	<0.001	0.72 (0.62–0.84)	<0.001	0.82 (0.68–0.99)	0.037
*Q4*	0.53 (0.44–0.63)	<0.001	0.56 (0.47–0.67)	<0.001	0.66 (0.51–0.84)	0.001
*P value for trend*		<0.001		<0.001		0.001
**Dietary OBS**						
Dietary OBS	0.97 (0.96–0.98)	<0.001	0.98 (0.97–0.98)	<0.001	0.98 (0.97–1.00)	0.008
Dietary OBS quartile						
*Q1*	ref		ref		ref	
*Q2*	0.84 (0.71–1.01)	0.066	0.87 (0.73–1.02)	0.088	0.90 (0.76–1.08)	0.262
*Q3*	0.66 (0.57–0.78)	<0.001	0.73 (0.63–0.86)	<0.001	0.80 (0.66–0.98)	0.027
*Q4*	0.56 (0.48–0.65)	<0.001	0.64 (0.55–0.75)	<0.001	0.71 (0.57–0.90)	0.005
*P value for trend*		<0.001		<0.001		0.004
**Lifestyle OBS**						
Lifestyle OBS	0.94 (0.90–0.98)	0.001	0.85(0.82–0.88)	<0.001	0.92 (0.88–0.96)	<0.001
Lifestyle OBS quartile						
*Q1*	ref		ref		ref	
*Q2*	0.98 (0.80–1.19)	0.814	0.73 (0.60–0.91)	0.004	0.86 (0.69–1.06)	0.154
*Q3*	0.99 (0.83–1.18)	0.913	0.74 (0.61–0.89)	0.002	0.91 (0.75–1.10)	0.314
*Q4*	0.78 (0.66–0.92)	0.003	0.50 (0.42–0.60)	<0.001	0.71 (0.59–0.84)	<0.001
*P value for trend*		0.002		<0.001		<0.001

CI – confidence interval, HR – hazard ratio, OBS – oxidation balance score, Q – quartile, ref – reference

*Model 1: crude model. Model 2: adjusted for sex, age, and race/ethnicity. Model 3: Adjusted for sex, age, race/ethnicity, education level, marital status, poverty income ratio, smoking status, history of cardiovascular disease, history of cancer, hyperlipidaemia, diabetes mellitus, and total energy intake.

### RCS analyses

**Figure 1:**
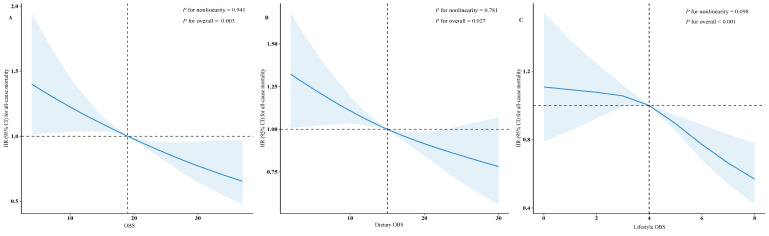
Restricted cubic spline analyses of the associations of OBS, dietary OBS, and lifestyle OBS with all-cause mortality in hypertensive individuals. Adjusted for sex, age, race/ethnicity, education level, marital status, poverty income ratio, smoking status, history of cardiovascular disease, history of cancer, hyperlipidaemia, diabetes mellitus, and total energy intake. **Panel A.** OBS. **Panel B.** Dietary OBS. **Panel C.** Lifestyle OBS. CI – confidence interval, HR – hazard ratio, OBS – oxidative balance score.

The weighted RCS analyses indicate a nearly linear dose-response relationship between OBS and its subscale scores and the risk of all-cause mortality (OBS: *P*-value for overall = 0.003, *P* for nonlinearity = 0.094; dietary OBS: *P*-value for overall = 0.027, *P*-value for nonlinearity = 0.781; lifestyle OBS: *P*-value for overall <0.001, *P* for nonlinearity = 0.098). The risk of all-cause mortality consistently decreased as OBS and its subscale scores increased (**Figure 1**), with a steeper decline in mortality risk observed when lifestyle OBS exceeded four points.

### Kaplan-Meier survival analyses

**Figure 2:**
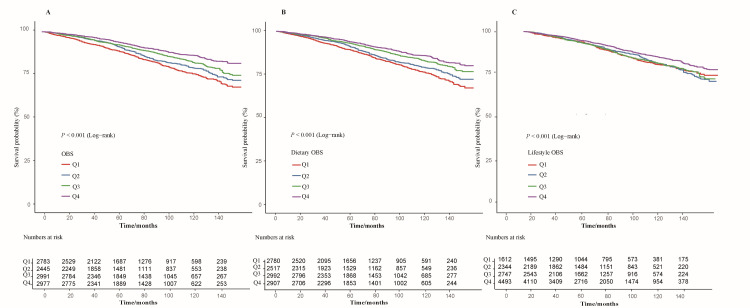
Kaplan-Meier survival analyses of OBS, dietary OBS, and lifestyle OBS with all-cause mortality in hypertensive individuals. **Panel A.** OBS. **Panel B.** Dietary OBS. **Panel C.** Lifestyle OBS. OBS – oxidative balance score, Q – quartile.

A total of 1764 deaths were recorded during a median follow-up of 73.4 months (interquartile range (IQR) = 40.3–110.2), corresponding to a weighted all-cause mortality rate of 12.23%. The median follow-up times for Q1, Q2, Q3, and Q4 were 69.6 months (IQR = 38.7–108.1), 72.7 months (IQR = 39.8–110.1), 73.0 months (IQR = 40.2–113.9), and 76.0 months (IQR = 42.4–108.0), respectively. The survival analysis demonstrated that individuals with higher OBS and its subscale scores had a significantly lower risk of all-cause mortality (log-rank *P*-value <0.001) (**Figure 2**).

### Stratified and sensitivity analyses

**Figure 3:**
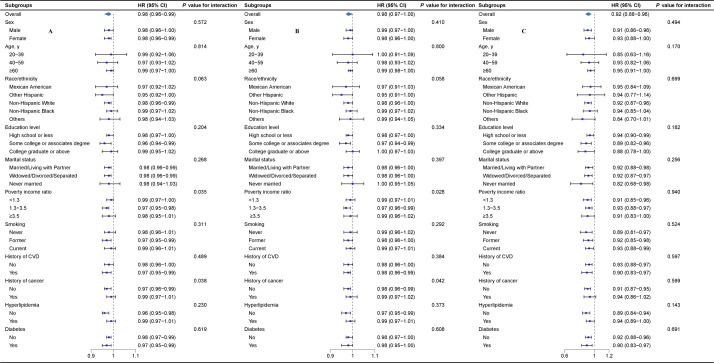
Stratified analyses of OBS, dietary OBS, and lifestyle OBS with all-cause mortality in hypertensive individuals. HRs and 95% CIs were calculated for each unit increase in OBS. Each stratification was adjusted for sex, age, race/ethnicity, education level, marital status, poverty income ratio, smoking status, history of cardiovascular disease, history of cancer, hyperlipidemia, diabetes mellitus, and total energy intake except the stratification factor itself. **Panel A.** OBS. **Panel B.** Dietary OBS. **Panel C.** Lifestyle OBS. CI – confidence interval, CVD – cardiovascular disease, HR – hazard ratio, OBS – oxidative balance score.

We found significant interactions between OBS and both PIR (*P*-value for interaction = 0.035) and cancer history (*P*-value for interaction = 0.038) in relation to the risk of all-cause mortality. The protective effect of OBS on all-cause mortality was more pronounced in individuals with a PIR of 1.3–3.5 (HR = 0.97; 95% CI = 0.95–0.98) and those without a history of cancer (HR = 0.97; 95% CI = 0.96–0.99) (**Figure 3**, Panel A). These findings may suggest effect modification rather than confounding. We noted similar results for dietary OBS (**Figure 3**, Panel B) and no significant interaction effects for lifestyle OBS (**Figure 3**, Panel C).

These findings remained stable in our sensitivity analysis after excluding participants who died within the first two years of follow-up (Table S2 in the **Online Supplementary Document**). In the fully adjusted model 3, participants in the highest OBS quartile had a 26% lower mortality risk compared to those in the lowest quartile (HR = 0.74; 95% CI = 0.57–0.97, *P* = 0.030). The results remained robust after excluding individuals with unreliable energy intake in the repeated analysis (Table S3 in the **Online Supplementary Document**). In the full adjusted analysis, participants in the highest OBS quartile exhibited a 30% lower mortality risk compared to those in the lowest quartile (HR = 0.70; 95% CI = 0.54–0.90, *P* = 0.006).

## DISCUSSION

In this large-scale prospective study using data from NHANES 2007–18, we investigated the relationship between OBS and all-cause mortality among individuals with hypertension. Our findings suggest that higher OBS levels, along with its dietary and lifestyle subscale scores, are associated with a significantly reduced risk of all-cause mortality. This association remained robust across various analytical models, as well as stratified and sensitivity analyses, highlighting the potential utility of OBS as a composite indicator for predicting survival outcomes in hypertensive individuals.

In recent years, OBS has been extensively studied to explore its impact on the onset and progression of various chronic diseases, including hypertension [8–10,29–33]. A cross-sectional study involving 317 US adults with hypertension found an inverse correlation between OBS and hypertension [34]. Another such study reported that higher OBS was negatively associated with the risk of resistant hypertension, arterial stiffness, and major adverse cardiovascular events [35]. A prospective cohort study in Korea showed that middle-aged and older adults with higher OBS levels had a lower risk of developing new-onset hypertension [36]. Prior research has also demonstrated that OBS serves as a reliable predictor of mortality among patients with cancer [37], non-alcoholic fatty liver disease [38], and metabolic syndrome [39].

Despite these contributions, we still lack a comprehensive understanding of the relationship between OBS and long-term prognosis among individuals with established hypertension. To our knowledge, this study is among the first to specifically evaluate the association between OBS and all-cause mortality in a large, nationally representative cohort of patients with hypertension. We found that individuals in the highest OBS quartile had a 34% lower risk of all-cause mortality compared to those in the lowest quartile, translating to an absolute mortality rate of 20 deaths per 1000 person-years in the highest OBS quartile, compared to 31 in the lowest quartile. Each unit increase in OBS was associated with a 2% reduction in mortality risk, even after adjusting for demographic, behavioural, and clinical covariates. Similarly, both dietary and lifestyle OBS subscales were independently associated with lower all-cause mortality, with participants in the highest quartiles for these subscales experiencing a 29% reduction in mortality risk relative to the lowest quartiles. Our findings suggest that OBS, as a composite measure reflecting the impact of dietary and lifestyle factors on oxidative balance, can effectively assess the mortality risk in hypertensive individuals.

Although the biological mechanism underlying the protective effect of a higher OBS on all-cause mortality in hypertensive patients has not been fully elucidated, it is possible that oxidative stress plays a critical role. Specifically, excessive production of ROS is the primary cause of oxidative stress, leading to endothelial dysfunction, arterial stiffness, atherosclerosis, cardiovascular remodelling, sympathetic nervous system activation, renal impairment, immune cell stimulation, and systemic inflammation, all of which are critical factors in the progression of hypertension and associated organ damage [9,40,41]. Dietary antioxidants such as fiber [42], vitamin C [43], vitamin E [44], carotenoids [45], and polyphenols [46] can neutralise excessive ROS and reduce oxidative stress. Regular physical activity can also enhance the body’s antioxidant capacity, improve cardiovascular function, and reduce cellular damage caused by oxidative stress [47,48]. In contrast, pro-oxidative factors like smoking [49], alcohol consumption [50,51], iron intake [52], and obesity [53,54] can elevate ROS production and exacerbate cell damage.

Our stratified analysis showed that the protective association between higher OBS and all-cause mortality was more evident among individuals with a PIR between 1.3 and 3.5. This group generally represents middle-income populations, which may benefit from greater economic stability, healthier behaviours, better healthcare access, and fewer competing health risks compared to those with lower income. We also observed that the inverse association between OBS and mortality was more pronounced among individuals without a history of cancer. In this subgroup, interventions aimed at improving oxidative balance may be more effective, likely due to the absence of cancer-related physiological burdens [55]. Conversely, individuals with a history of cancer are likely to experience persistent oxidative stress and chronic inflammation resulting from both the malignancy itself and treatments such as chemotherapy and radiation therapy [56–58]. These factors may attenuate the potential benefits of antioxidant-promoting behaviours. Additionally, differences in disease progression, treatment timelines, and survivorship care may also play a role and should be explored in future research.

Considering the high prevalence of hypertension and its substantial global burden of mortality, our findings have significant implications for both public health and clinical practice. Promoting dietary patterns rich in antioxidants, such as the Mediterranean diet or the ‘Dietary Approaches to Stop Hypertension’ diet, may improve oxidative balance and reduce mortality risk among hypertensive patients. Public health campaigns should prioritise increasing the accessibility and affordability of vegetables, whole grains, fruits, and nuts. Additionally, encouraging smoking cessation, physical activity, and maintaining a healthy weight should be central to hypertension management strategies, as these behaviours not only improve oxidative balance, but also address risk factors for other chronic diseases. Moreover, the OBS may serve as a practical tool for assessing oxidative balance and identifying individuals at higher risk of mortality, enabling clinicians to leverage OBS to guide personalised interventions and optimise dietary and lifestyle factors. However, before OBS can be implemented in clinical settings, prospective validation and feasibility studies are needed to standardise its calculation, evaluate its clinical utility, and determine its cost-effectiveness.

Our study has several strengths, including the use of a large, nationally representative dataset, a comprehensive assessment of dietary and lifestyle factors, and rigorous adjustment for potential confounders. However, several limitations should also be acknowledged. First, the observational study design prevented us from establishing causal relationships. Second, NHANES lacked longitudinal data on OBS, and reliance on baseline values may not capture dynamic changes in diet and lifestyle during follow-up. Third, dietary recalls and self-reported behavioural data may be susceptible to recall and social desirability bias, particularly for alcohol and smoking, consequently inflating or deflating exposure estimates, as well as affecting the OBS accuracy and its association with mortality. Fourth, despite adjusting for numerous covariates, residual confounding remains possible, especially from unmeasured factors such as psychosocial stress, sleep patterns, medication adherence, and healthcare access. Fifth, as the study included only adults from the USA, the findings may not be generalisable to low- and middle-income countries with different hypertension prevalence, healthcare infrastructure, or lifestyle patterns. This study also did not sufficiently address implications or challenges in low-resource settings where nutritional and lifestyle interventions are more complex. Finally, the observed interactions between OBS and PIR or cancer history should be interpreted with caution due to potentially small subgroup sizes and multiple testing issues. The influence of socioeconomic status and comorbidities on these associations warrants further investigation in more diverse and larger populations.

## CONCLUSIONS

Our findings suggests that higher OBS and its subscale scores are significantly associated with a lower risk of all-cause mortality among individuals with hypertension. While this highlights the importance of maintaining a favourable oxidative balance, future longitudinal and interventional studies are needed to validate these associations, elucidate underlying mechanisms, and evaluate the potential utility of OBS in clinical and public health practice.

## Additional material


Online Supplementary Document
