# Supplementary material for: Oxidative balance score and all-cause mortality among hypertensive individuals
Source: J Glob Health. 2025 Nov 14;15:04285. doi: 10.7189/jogh.15.04285 (PMC12615001; doi:10.7189/jogh.15.04285)
Supplement: Online Supplementary Document [file jogh-15-04285-s001.pdf]

Supplement to: Huang L, Zhang H, Qin M, Ni Z, Huang W, Li J, Sheng L, Guo L, Zhan J. Oxidative balance score and all-cause mortality among hypertensive individuals. J Glob Health. 2025;15.04285.

Supplementary Data

Figure S1. Flowchart of study participants

Table S1. Oxidative balance score assignment scheme

Table S2. Association of OBS with all-cause mortality after excluding participants who died within two years of follow-up (n = 10 845)

Table S3. Association of OBS with all-cause mortality after excluding individuals with unreliable energy intake (n = 10 562)

Table S4. Outline of JoGH's Guidelines for Reporting Analyses of Big Data Repositories Open to the Public (GRABDROP) items

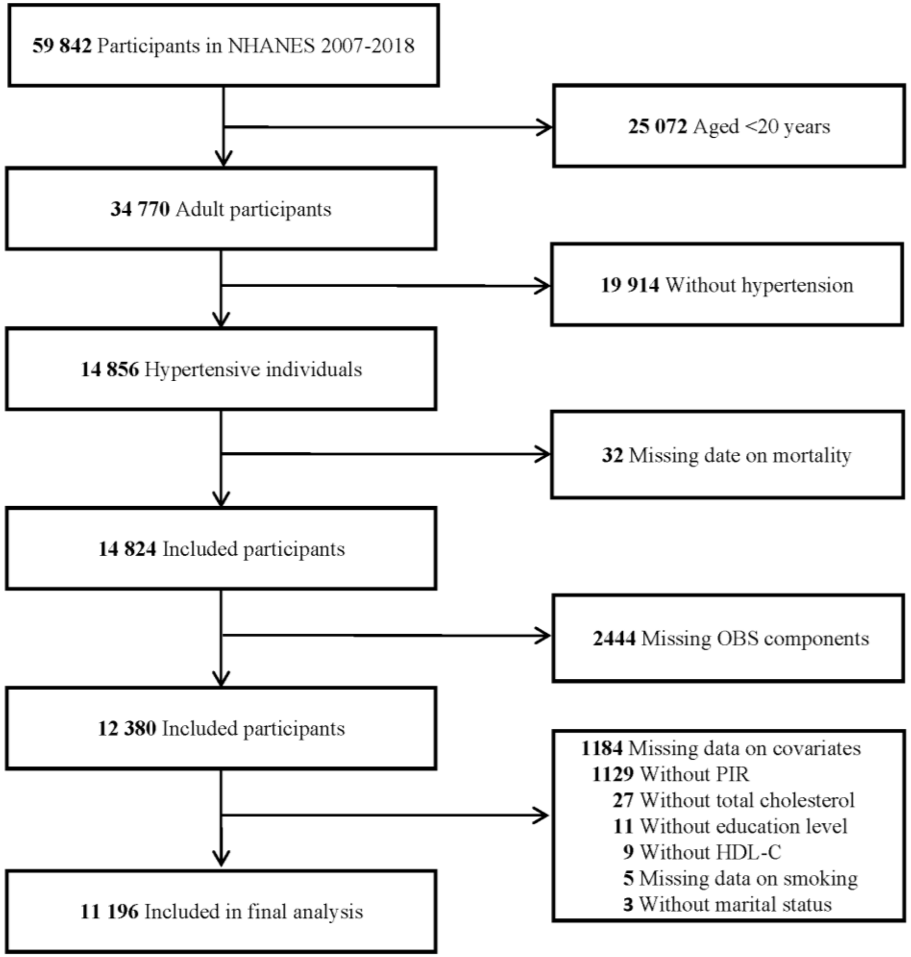

Figure S1. Flowchart of study participants. NHANES – National Health and Nutrition Examination Survey, OBS – oxidative balance score, PIR – poverty income ratio,

HDL-C – high-density lipoprotein cholesterol.

Table S1. Oxidative balance score assignment scheme

OBS components	Property	Male			Female		
		0	1	2	0	1	2
Dietary OBS components							
Dietary fiber (g/d)	A	<12.4	12.4–20.5	≥20.5	<10.2	10.2–16.7	≥16.7
Carotene (RE/d)	A	<74.09	74.09–266.39	≥266.39	<67.34	67.34–272.33	≥272.33
Riboflavin (mg/d)	A	<1.78	1.78–2.69	≥2.69	<1.33	1.33–2.01	≥2.01
Niacin (mg/d)	A	<21.61	21.61–32.55	≥32.55	<14.98	14.98–22.44	≥22.44
Vitamin B6 (mg/d)	A	<1.63	1.63–2.55	≥2.55	<1.14	1.14–1.83	≥1.83
Total folate (mcg/d)	A	<304.68	304.68–487.29	≥487.29	<233.49	233.49–376.75	≥376.75
Vitamin B12 (mcg/d)	A	<3.26	3.26–6.19	≥6.19	<2.31	2.31–4.44	≥4.44
Vitamin C (mg/d)	A	<31	31–89.8	≥89.8	<27.68	27.68–81.99	≥81.99
Vitamin E (ATE) (mg/d)	A	<6.11	6.11–10.42	≥10.42	<4.67	4.67–8.12	≥8.12
Calcium (mg/d)	A	<692.92	692.92–1164.20	≥1164.20	<588.88	588.88–940.86	≥940.86
Magnesium (mg/d)	A	<252.95	252.95–371.90	≥371.90	<198.27	198.27–283.79	≥283.79
Zinc (mg/d)	A	<9.29	9.29–14.68	≥14.68	<6.81	6.81–10.37	≥10.37
Copper (mg/d)	A	<1.01	1.01–1.51	≥1.51	<0.80	0.80–1.18	≥1.18
Selenium (mcg/d)	A	<96.7	96.7–146.7	≥146.7	<68.7	68.7–104.5	≥104.5
Total fat (g/d)	P	≥107.24	69.77–107.24	<69.77	≥78.66	50.45–78.66	<50.45
Iron (mg/d)	P	≥18.23	11.96–18.23	<11.96	≥13.91	8.98–13.91	<8.98
Lifestyle OBS components							
Physical activity (MET–minute/week)	A	<500	500–1000	≥1000	<500	500–1000	≥1000
Alcohol (g/d)	P	≥30	0–30	0	≥15	0–15	0
Body mass index (kg/m2)	P	≥30	25–30	<25	≥30	25–30	<25
Cotinine (ng/mL)	P	≥0.31	0.02–0.31	<0.02	≥0.07	0.01–0.07	<0.01

OBS – oxidation balance score, A – antioxidant, P – pro-oxidant, RE – retinal equivalent, ATE – alpha-tocopherol equivalent, MET – metabolic equivalent.

Table S2. Association of OBS with all-cause mortality after excluding participants who died within two years of follow-up (n = 10 845)

Model	Model 1		Model 2		Model 3	
	HR (95% CI)	*P* value	HR (95% CI)	*P* value	HR (95% CI)	*P* value
OBS	0.97 (0.96–0.98)	<0.001	0.97 (0.96–0.98)	<0.001	0.98 (0.97–1.00)	0.010
OBS quartile						
Q1	Ref		Ref		Ref	
Q2	0.89 (0.74–1.07)	0.215	0.85 (0.71–1.01)	0.006	0.94 (0.77–1.15)	0.553
Q3	0.70 (0.61–0.81)	<0.001	0.73 (0.63–0.85)	<0.001	0.86 (0.70–1.04)	0.121
Q4	0.57 (0.47–0.68)	<0.001	0.61 (0.51–0.72)	<0.001	0.74 (0.57–0.97)	0.030
P for trend		<0.001		<0.001		0.020

OBS – oxidation balance score, HR – hazard ratio, CI – confidence interval, Q – quartile, Ref – reference.

Model 1: Crude model.

Model 2: Adjusted for sex, age, and race/ethnicity.

Model 3: Adjusted for sex, age, race/ethnicity, education level, marital status, poverty income ratio, smoking status, history of cardiovascular disease, history of cancer, hyperlipidemia, diabetes mellitus, and total energy intake.

Table S3. Association of OBS with all-cause mortality after excluding individuals with unreliable energy intake (n = 10 562)

Model	Model 1		Model 2		Model 3	
	HR (95% CI)	*P* value	HR (95% CI)	*P* value	HR (95% CI)	*P* value
OBS	0.97 (0.96–0.98)	<0.001	0.97 (0.96–0.98)	<0.001	0.98 (0.97–0.99)	0.001
OBS quartile						
Q1	Ref		Ref		Ref	
Q2	0.87 (0.73–1.05)	0.144	0.81 (0.67–0.97)	0.019	0.88 (0.73–1.07)	0.205
Q3	0.71 (0.60–0.82)	<0.001	0.72 (0.62–0.84)	<0.001	0.85(0.71–1.02)	0.076
Q4	0.57 (0.48–0.68)	<0.001	0.56 (0.47–0.67)	<0.001	0.70 (0.54–0.90)	0.006
P for trend		<0.001		<0.001		0.006

OBS – oxidation balance score, HR – hazard ratio, CI – confidence interval, Q – quartile, Ref – reference.

Model 1: Crude model.

Model 2: Adjusted for sex, age, and race/ethnicity.

Model 3: Adjusted for sex, age, race/ethnicity, education level, marital status, poverty income ratio, smoking status, history of cardiovascular disease, history of cancer, hyperlipidemia, diabetes mellitus, and total energy intake.

Table S4. Outline of JoGH's Guidelines for Reporting Analyses of Big Data Repositories Open to the Public (GRABDROP) items

1. Please list all papers published by each co-author in previous three years that were based on secondary analysis of a big data repository	The following studies based on NHANES data were conducted by the authors:<br>Huang LZ, Ni ZB, Yao QR, Huang WF, Li J, Wang YQ, Zhang JY. Association of pan-immune-inflammatory value with metabolic dysfunction-associated steatotic liver disease: findings from NHANES 2017-2020. BMC Gastroenterol. 2025 Jan 3;25(1):4.<br>Huang LZ, Ni ZB, Huang WF, Sheng LP, Wang YQ, Zhang JY. Association between cardiovascular health and metabolic dysfunction-associated steatotic liver disease: a nationwide cross-sectional study. J Health Popul Nutr. 2025 Jan 13;44(1):9.
2. Please explain the key elements of your study design and the use of the available datasets that make your study an original scientific contribution	This study utilized data from the National Health and Nutrition Examination Survey spanning 2007 to 2018 to investigate the association between the oxidative balance score (OBS) and all-cause mortality among individuals with hypertension. To the best of our knowledge, this represents the first comprehensive analysis of this relationship specifically within a hypertensive population. The study design employs rigorous statistical methods—including Cox proportional hazards models, restricted cubic spline analyses, and a series of stratified and sensitivity analyses—to enhance the robustness and reliability of the findings. The novel application of OBS in this clinical context highlights the study's originality and contributes valuable insights to the field of cardiovascular and metabolic epidemiology.
3. Please list all publications that addressed similar research questions in the same dataset and indicate where you cited them in your paper	One relevant publication was identified: Zhang YJ et al., "Alcohol drinking triggered decrease of oxidative balance score is associated with high all-cause and cardiovascular mortality in hypertensive individuals: findings from NHANES 1999-2014", cited as reference 50 in our manuscript.
4. Please explain how you addressed multiple testing through an appropriately rigorous statistical threshold and indicate this in the methods section	We employed Bonferroni adjustment to account for multiple testing using a two-sided *P*-value of 0.05, which resulted in an adjusted significance level of *P* =

	0.017 (0.05/3).
5. Please declare to what extent have AI chatbots been used in developing your paper and to which parts of the paper did they contribute	We declare that AI chatbots were not used at any stage in the development of this manuscript.

Adapted from: Rudan I, Song P, Adeloye D, Campbell H. Journal of Global Health's Guidelines for Reporting Analyses of Big Data Repositories Open to the Public (GRABDROP): preventing 'paper mills', duplicate publications, misuse of statistical inference, and inappropriate use of artificial intelligence. J Glob Health. 2025;15:01004.
